# The influence of filler type on the separation properties of mixed-matrix membranes

**DOI:** 10.1007/s11696-017-0363-9

**Published:** 2017-12-15

**Authors:** Małgorzata Gnus, Gabriela Dudek, Roman Turczyn

**Affiliations:** Department of Physical Chemistry and Technology of Polymers, Faculty of Chemistry, Silesian University of Technology, M. Strzody 9 Street, 44-100 Gliwice, Poland

**Keywords:** Pervaporation, Composite membranes, Metal oxides

## Abstract

Chitosan-based membranes filled with different metal oxide particles were prepared and their performance in ethanol dehydration process depending on the type of oxide and loading was discussed. For membrane preparation three oxides: TiO_2_, Cr_2_O_3_ or Fe_3_O_4_ were selected. From experimental data suitable ethanol and water transport coefficients were evaluated. As shown in the results, applied fillers in different ways affect the separation properties. Presence of TiO_2_ significantly affects the normalized total flux, increasing its value. On the other hand, addition of Fe_3_O_4_ influences most of all the separation factor, which is the among all investigated membranes. For membranes containing chromium(III) oxide as a filler, improvement in the separation properties is observed only in the case when the Cr_2_O_3_ content equals to 5 wt%. Above this concentration significant deterioration of separation properties is observed. The best performance has mixed-matrix membranes (MMMs) with magnetite, where the values of PSI are equal to 16.3 and 296.8 kg/m^−2^ h µm for pristine and 15 wt% filler content, respectively.

## Introduction

Membrane separation techniques attract much attention in many fields, since they offer high efficiency with energy savings and have been an environmentally friendly method to deliver the aforementioned properties with the minimal increase in space and weight. Otherwise, pervaporation is, among them, one of the membrane separation techniques applicable for multi-component solutions. Especially, pervaporation is a promising technique for separation of organic liquid mixtures such as azeotropic mixtures (Bolto et al. 2011; Chapman et al. 2008). The success of pervaporation depends mainly on the nature of the polymeric membrane chosen for a particular application in addition to its physical state, structure, chemically interacting groups, addition of filler particles, physicochemical properties of the separated feed mixture, as well as feed component–component and component–membrane interactions (Hussain et al. 1996). However, the key to success in pervaporation separation lies in the development of a suitable membrane material that offers high flux, good separation factor (selectivity) and long-term stability, and also favourable mechanical strength to withstand the cyclic modes of operating conditions.

Chitosan, as a natural linear biopolyaminosaccharide, is obtained by alkaline deacetylation of chitin and is its most important derivative (Crini and Badot 2008; Dutta et al. 2004). Unfortunately, membranes prepared only with chitosan posed a lack of mechanical strength and stability mainly due to excessive swelling in aqueous solutions. This disadvantage could be overcome by chemical or physical stabilization of membrane structure. The chemical modification of chitosan by using crosslinking reaction offers an alternative pathway for producing chemically more stable chitosan derivatives, which can extend the potential applications of this biopolymer to more areas. The application of chitosan membranes in pervaporation process has played an important role. They have been intensively studied and developed in numerous research groups, and widely used in a plain or modified form Baig 2008; Baker 2004; Chen et al. 2007; Ge et al. 2000; Kang et al. 2013; Lee and Shin 1991; Lee et al. 1992; Sun et al. 2008; Sunitha et al. 2012; Uragami and Takigawa 1990; Wu et al. 2016; Yang et al. 2009; Zhang et al. 2007; and Zielinska et al. 2011.

In recent years, the trend has shifted more towards developing filler reinforced matrices as pervaporation membranes (Sun et al. 2008; Yang et al. 2009; Kang et al. 2013; Zhao et al. 2014; Wu et al. 2016). Incorporation of different micro- or nano-sized materials—metal oxides (Balta et al. 2012; Jiang et al. 2007; Li et al. 2012; Ng et al. 2013; Rybak et al. 2014; Thamaphat et al. 2008; Yang et al. 2009; Zhao et al. 2014), zeolites (Khoonsap and Amnuaypanich 2011; Nigiz and Hilmioglu 2013; Ong et al. 2016; Premakshi et al. 2015; Zhou et al. 2016), silica (Araki et al. 2016, Tancharernrat et al. 2014; Xia et al. 2016)—with polymer matrix, when both of them have different flux and selectivity provides the possibility to obtain synergistic effects and better designing a membrane with desired properties, so hydrophilic polymeric materials filled with the nano-sized fillers could be the ideal materials to selectively separate water from its mixture with an organic component.

In principle, the incorporation of the inorganic component can be seen as a relatively easy modification of existing methods for fabricating large-surface area polymeric membranes. Therefore, mixed-matrix membranes (MMMs) possess an economic advantage over inorganic membranes.

In our previous research (Dudek et al. 2014a, b; Turczyn et al. 2015; Gnus et al. 2015), we investigated chitosan membranes with different addition of iron oxide nanoparticles in the process of ethanol dehydration. The results showed that the addition of particles to chitosan matrix created extra free volumes in polymer, and in consequence, offered space for water molecules to permeate easier through the membranes.

The aim of this work was the comparison between different metal oxide fillers and examination of their influence on the separation properties of chitosan MMMs used for the pervaporative dehydration of ethanol–water solutions.

## Theoretical

For evaluation of the membrane efficiency, several parameters are evaluated. First of all is flux, and it is determined by the amount of permeate collected over a given period of time. The permeation flux *J* of component *i* is calculated using the following equation:

1 $$ J_{i} = \frac{{m_{i} }}{A\;t}\;\left[ {\frac{\text{kg}}{{{\text{m}}^{2} \;{\text{h}}}}} \right], $$

where *m*_*i*_ is the weight of component *i* in permeate [kg], *A* is the effective membrane area [m^2^], and *t* is the permeation time [h].

Flux could be also normalized to the equal thickness of 1 μm and calculated as the normalized flux of component *i*:

2 $$ J_{{{\text{N}}i}} = \frac{{J_{i} }}{d}\;\left[ {\frac{\text{kg}}{{{\text{m}}^{ 2} \;{\text{h}}\;\mu {\text{m}}}}} \right], $$

where *d* is the membrane thickness [μm].

To designate the permeability coefficient, first it was necessary to determine the volume of the individual components in the collected permeate.

3 $$ V_{i} = \frac{{n_{i} RT}}{{p_{i} }}\;[{\text{m}}^{3} ], $$

where *n*_*i*_ is the amount of component *i* [mol], *R* is the gas constant, 8.315 [J/mol K], *T* is the temperature of measurement [K], and *p*_*i*_ the partial pressure of component *i* [Pa].

Next, it is necessary to determine the measurable flow of the component *i*:

4 $$ Q_{i} = \frac{{V_{i} }}{t}\;\left[ {\frac{{{\text{m}}^{ 3} }}{\text{s}}} \right], $$

where *V*_*i*_ is the volume of component *i* [m^3^] and *t* is the collection time [s].

Then, to make the flow of individual components independent from parameters such as temperature and pressure, it was standardized according to the following equation:

5 $$ Q_{\text{STP}} = Q_{i} \frac{{T_{\text{STP}} p}}{{p_{\text{STP}} T}}\;\;\left[ {\frac{{{\text{m}}_{\text{STP}}^{3} }}{\text{s}}} \right], $$

where *T*_STP_ is the standard temperature 298,15 K, *T* is the temperature of measurement [K], $p_{\text{STP}}$ the standard pressure 101,300 Pa, and *p* is the ambient pressure [Pa].

In the last step diffusive flux of component *i* is evaluated:

6 $$ J_{{{\text{D}}i}} = \frac{{Q_{\text{STP}} }}{A}\;\left[ {\frac{{{\text{m}}_{\text{STP}}^{3} }}{{{\text{m}}^{2} \,{\text{s}}}}} \right], $$

where *Q*_STP_ is the flow in standard conditions [$m_{\text{STP}}^{3} /{\text{s}}$], *A*—active surface of membrane [m^2^].

Finally, when the diffusive flux and partial vapour pressure of component *i* in a feed are known, it is possible to estimate the permeability coefficient of component *i*:

7 $$ P_{i} = J_{{{\text{D}}i}} \frac{d}{\Delta p}\;\left[ {\frac{{{\text{m}}_{\text{STP}}^{3} \,{\text{m}}}}{{{\text{m}}^{2} \,{\text{s}}\,{\text{mmHg}}}}} \right], $$

where *J*_Di_ is the diffusive flux of component *i* [${\text{m}}_{\text{STP}}^{ 3} / {\text{m}}^{ 2}$ s], *d*is the membrane thickness [m], and Δ*p* is the difference between partial vapour of component *i* in feed and pressure on the permeate side [mmHg].

Diffusion coefficients of individual components of the mixture were designated by “time lag” method. For evaluation the time delay of component *i*, plot the cumulative dependency curves of the component collected mass from the time of the process and designate the tangent to the straight line formed on the curve—corresponding to the established process.

**Fig. 1:**
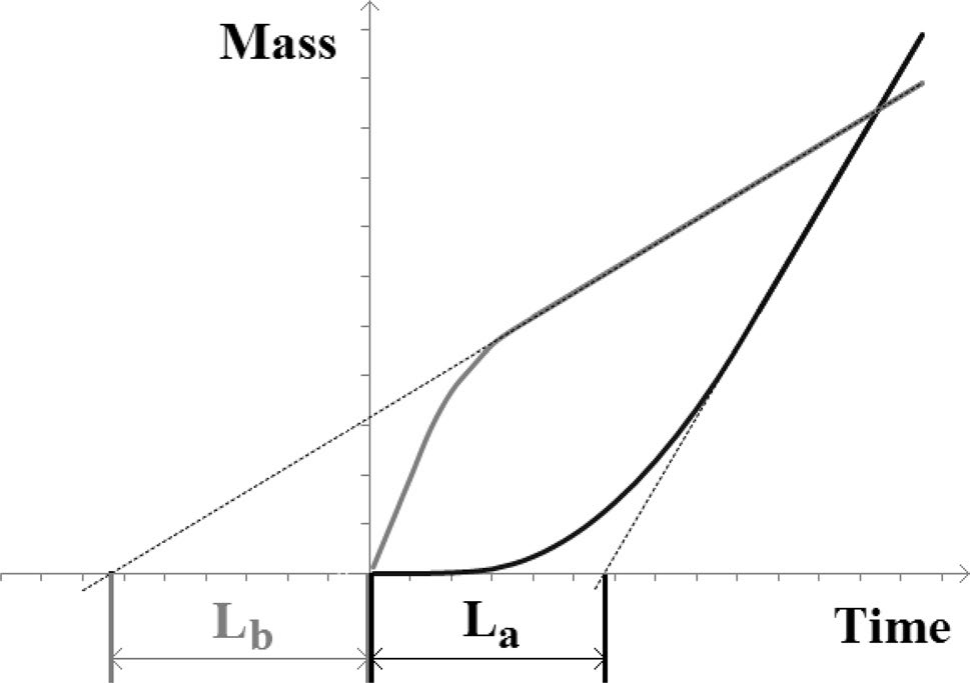
The methodology of the “time lag” determination. Depending on the initial state of membrane, the curve may have two different shapes and two corresponding time lags *L*_a_ or *L*_b_ could be derived

Depending on the initial state, the curve may have a different shape (Fig. 1) and diffusion coefficient could be calculated by two ways using *L*_a_ or *L*_b_, respectively:

8 $$ D_{{{\text{A}}i}} = \frac{{d^{2} }}{{6L_{\text{a}} }}\left[ {\frac{{{\text{m}}^{ 2} }}{\text{s}}} \right]\;{\text{or}}\;D_{{{\text{B}}i}} = - \frac{{d^{2} }}{{3L_{\text{b}} }}\left[ {\frac{{{\text{m}}^{ 2} }}{\text{s}}} \right], $$

where *d* is the membrane thickness [m], *L*_a_ and *L*_b_ the time lag [s] determined from the mass versus time graph.

Knowing the permeability and diffusion coefficients, it is possible to estimate the solubility coefficient of component *i*:

9 $$ S_{i} = \frac{{P_{i} }}{{D_{i} }}\;\left[ {\frac{{{\text{m}}_{\text{STP}}^{ 3} }}{{{\text{m}}\,{\text{mmHg}}}}} \right]. $$

For describing the separation properties of the membrane, separation factor (αAB) was used. Separation factor determines the separation of mixture through the changes of component concentration in the feed and permeate and is calculated by:

10 $$ \alpha_{\text{AB}} = \frac{{y_{\text{A}} /y_{\text{B}} }}{{x_{\text{A}} /x_{\text{B}} }}, $$

where *x*_A_, *x*_B_ is the weight fraction of component in the feed [wt%], *y*_A_, *y*_B_ the weight fraction of components in permeate [wt%].

In order to compare the separation efficiency of different investigated membranes, a pervaporation separation index, PSI expressed by following equation is used:

11 $$ {\text{PSI}} = J_{N} (\alpha_{\text{AB}} - 1)\;\left[ {\frac{\text{kg}}{{{\text{m}}^{2} \;{\text{h}}\;\mu {\text{m}}}}} \right], $$

where *J*_*N*_ is the normalized total permeate flux [kg/m^2^ h μm], and *α*_AB is the_ separation factor.

## Experimental

### Chemicals

Chitosan (*M*_n_ = 600–800 kDa; Acros Organics), iron(II) chloride hexahydrate (pure for analysis, POCh), iron(III) chloride anhydrous (pure, POCh), sodium hydroxide (pure, POCh), 2,2′-(ethylenedioxy)bis(ethylamine) (98%, Aldrich), 25% ammonia solution (pure for analysis, Chempur) glycidyl chloride (99%, Acros Organics), titanium(IV) oxide and chromium(III) oxide (pure, POCh), glacial acetic acid (pure for analysis, POCh).

### Preparation of iron(II,III) oxide

Magnetite was prepared by coprecipitation method. Briefly, 6 g FeCl_2_·4H_2_O and 7.4 g FeCl_3_ were separate dissolved in bakers containing 40 ml of distilled water. The solutions were combined in larger baker, 5.8 ml of EDBE [2,2′-(ethylenedioxy)bis(ethylamine)] was added and mixed on a magnetic stirrer for about 5 min. Then slowly added 50 ml of NH_3(aq)_ (25%) and stir for 1 h until the reaction was completed. After that, baker was placed on a strong magnet to speed up the sedimentation of the resulting magnetite particles and poured out the supernatant. Next, 50 ml of 5% NH_3(aq)_ with 5.8 ml of EDBE was added and mixed. The suspension was heated for few minutes, then decantation of obtained powder was used. Then obtained magnetite powder was filtered off and washed with hot distilled water until a negative silver test for the presence of Cl^−^ in filtrate and dried.

### Characterization of used particles

X-ray powder diffraction (XRD) patterns of the samples were recorded on a X-Pert Philips PW 3040/60 diffractometer operating at 30 mA and 40 kV. The radiation wavelength (λCu Kα) was 1.54056°. The patterns were recorded in a 2*θ* range from 5° to 110°.

Dynamic light-scattering (DLS) experiments were performed on a particle-size analyzer, model Nano ZS90 (Malvern instruments, UK). All the measurements were carried out at a scattering angle of 90 and a temperature of 25 °C, which was controlled by means of a thermostat. A dilute solution of the sample was prepared by dispersing the powder in ethylene glycol.

### Membranes preparation

**Fig. 2:**
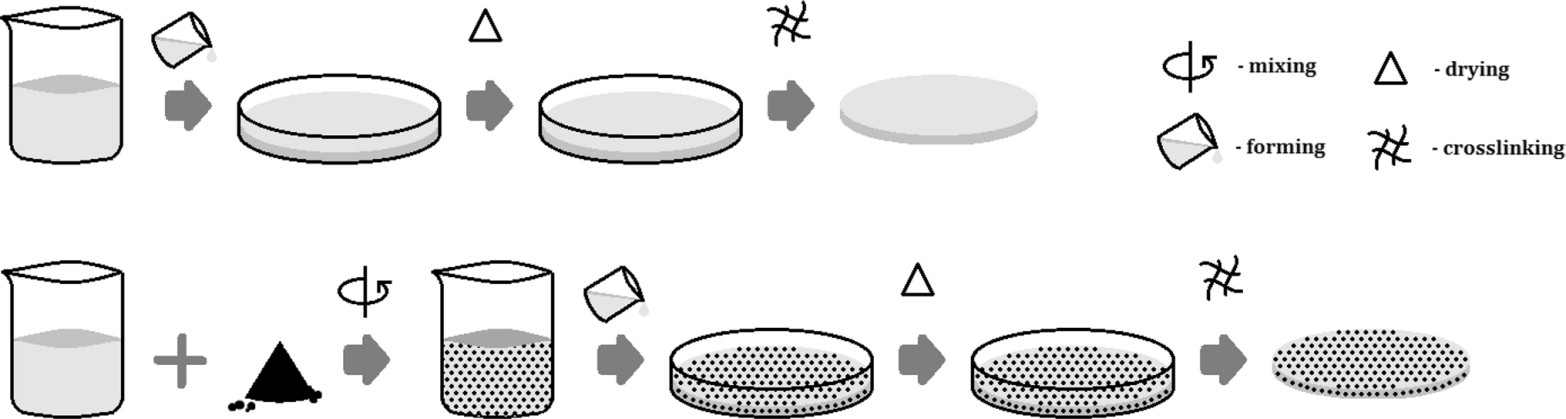
Scheme of membrane preparation

Membranes were prepared by solution casting and solvent evaporation technique. The prepared 3 wt% chitosan solution in 1 vol% acetic acid. This solution was mixed with an appropriate portion of metal oxide particles, micro-sized Cr_2_O_3_ as well as nano-sized TiO_2_ or Fe_3_O_4_ (5; 10; 15 wt% based on the dry weight of the polymer matrix), was casted into a 16 cm diameter Petri dish and left until solvent evaporation at 40 °C. Next, chitosan membranes were immersed in 0.08 M glycidyl chloride in 2% sodium hydroxide solution by 24 h at room temperature. After this time membranes were subsequently washed with distilled water until obtained neutral pH and dried in room temperature. The pristine chitosan membrane was prepared in the same manner except for the addition of metal oxide filler (see Fig. 2).

The membrane thickness was measured using waterproof precise coating thickness gauge MG-401 ELMETRON, estimated as a mean values of at least 25 measurements.

### Pervaporation

**Fig. 3:**
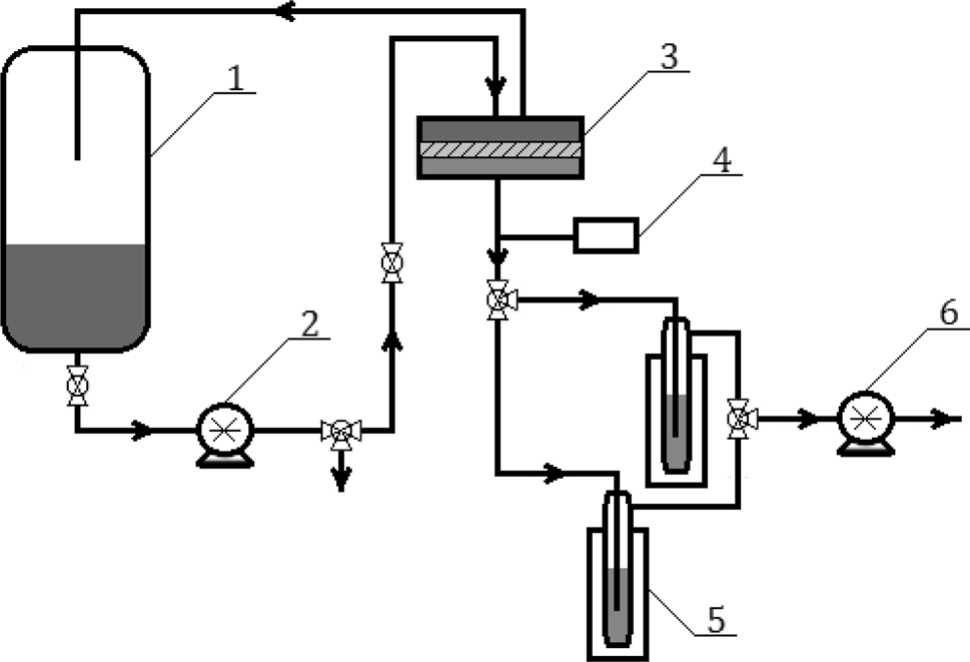
Scheme of pervaporation setup: 1—feed tank, 2—circulation pump, 3—separation chamber, 4—vacuum gauge, 5—cooled collection traps, 6—vacuum pump

Pervaporation experiments were performed at room temperature on pervaporation setup (Fig. 3). Prepared membrane was placed in a membrane chamber (3) with effective membrane area 10.39 × 10^−3^ m^2^. Measurements were performed for membranes in contact with solution containing 96 wt% of ethanol. Mixture (1 dm^3^) at room temperature was poured into the feed tank (1) and pumped, using a circulation pump (2) with velocity 9.25 × 10^−2^ m^3^/h, to the membrane chamber where feed was separated. Next, retentate was recirculated to the feed tank; however, permeate vapours were condensed in a liquid nitrogen cooled trap (5). Permeate was collected for duration of 7.5 h and weighed after defrosting on analytical balance to determine the value of total flux. The reduced pressure on the permeate side was 350–390 Pa was produced by a vacuum pump (6) and controlled with a vacuum gauge (4).

Before measurements, each membrane was conditioned for a few minutes in a membrane chamber being in contact with circulating feed solution and after applying of reduced pressure and its stabilization, flux measurement was started.

The collected samples of permeate as well as feed (before and after process) were analysed by gas chromatography technique. Measurements were performed on a gas chromatograph (Agilent Technologies 6850 Network GC System) equipped with an Elite-WAX ETR column (30 m), and FID detector. Measurements were carried out with nitrogen as carrier gas, with 1 μl sample injection and measurement time of 6 min at constant temperature − 80 °C. The ethanol content was determined on the basis of the prepared calibration curve.

### Degree of swelling

The membrane swelling test in water was determined by weight method. The piece of membrane was weighting before and after their immersion for 24 h in distilled water or ethanol (96%). Mass change of analysed membranes was measured using analytical balance and degree of swelling was calculated from following equation:

12 $$ {\text{DS}} = \frac{{W_{\text{wet}} - {\kern 1pt} \,\;W_{\text{dry}} }}{{W_{\text{dry}} }} \times 100\;[\% ], $$

where *W*_wet_ is the weight of the swollen membrane [g] and *W*_dry_ the weight of the dried membrane samples [g].

## Results and discussion

**Fig. 4:**
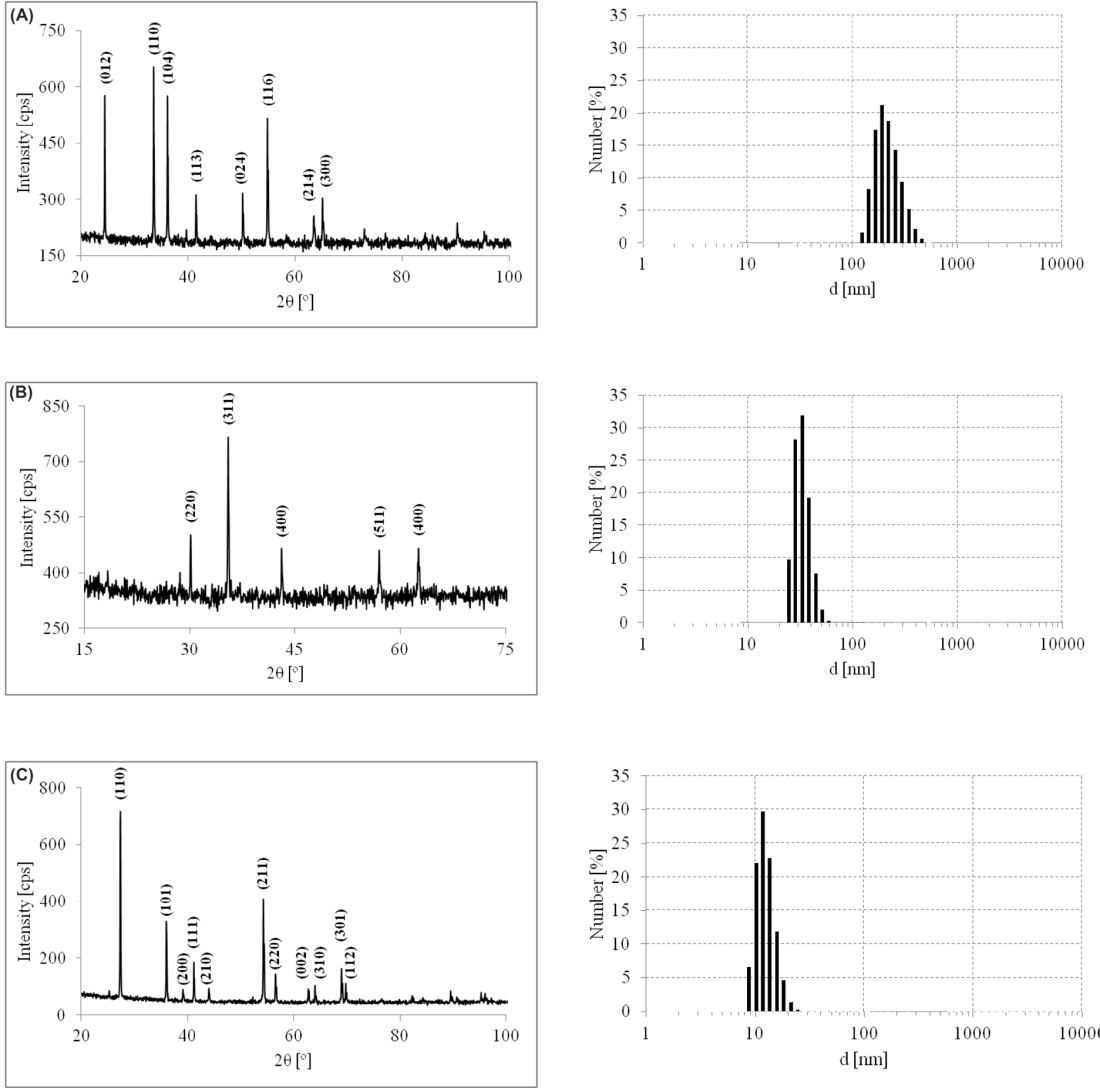
XRD and DLS analysis of the used particles: Cr_2_O_3_ (**a**), Fe_3_O_4_ (**b**) and TiO_2_ (**c**)

The phase composition and size distribution of used particles were characterized by power X-ray diffraction (XRD) and differential light scattering (DLS), respectively. Figure 4a shows the XRD pattern of the Cr_2_O_3_. The major peaks were indexed as (012), (110), (104), (113), (024), (214) and (300) which are consistent with references (Farzaneh and Najafi 2011). DLS histogram shows that the grain size is within the 200 nm limit. Figure 4b shows the XRD of the magnetite nanoparticles prepared by coprecipitation method. The observed peaks: (220), (311), (400), (511) and (400) planes confirm the Fe_3_O_4_ spinel structure (Pati et al. 2012, Han et al. 2014). According to DLS measurement, the grain size is within the 25–50 nm range. The TiO_2_ was confirmed by presence of peaks: (110), (101), (200), (111), (220), (002), (310), (301) and (112) planes corresponding to rutile phase (Fig. 4c), but X-ray line shape could suggest that it has micro-sized grains (Thamaphat et al. 2008). On the other hand, DLS measurement shows that particles hydrodynamic diameter was 9–12 nm. This is probably due to the good stability of dispersion in ethylene glycol particles, which limits aggregation of TiO_2_ particles.

The evaluated parameters for pervaporation process describing transport properties, i.e. diffusion, permeation and solubility coefficients of ethanol and water through both, pristine and composite epichlorohydrin-crosslinked chitosan membranes are collected in Table 1.

**Table 1:** Evaluated transport parameters of water and ethanol of epichlorohydrin-crosslinked chitosan membranes without and with different amount of inorganic fillers

Membrane	CS	CS_Cr_2_O_3_			CS_Fe_3_O_4_			CS_TiO_2_		
Filler content [wt%]	0	5	10	15	5	10	15	5	10	15
Water										
Diffusion coefficient, *D* × 10^14^[m^2^/s]	8.9	37.2	20.1	4.4	101.2	162.4	39.8	47.3	6.7	13.4
Permeation coefficient, *P* × 10^12^ [${\text{m}}_{\text{STP}}^{ 3}$ m/m^2^ mmHg s]	34.9	44.5	38.3	37.2	28.5	32.0	31.1	35.4	45.1	31.9
Solubility coefficient, *S* [${\text{m}}_{\text{STP}}^{ 3}$ /m mmHg]	392.25	119.60	190.60	884.77	28.19	19.69	78.19	74.82	673.28	238.13
Ethanol										
Diffusion coefficient, *D* × 10^14^[m^2^/s]	131.2	464.1	58.7	67.4	70.1	50.7	22.7	99.7	38.3	506.4
Permeation coefficient, *P* × 10^12^ [${\text{m}}_{\text{STP}}^{ 3}$ m/m^2^ mmHg s]	5.2	2.7	9.4	4.5	6.2	6.8	4.8	4.8	7.8	7.1
Solubility coefficient, *S* [${\text{m}}_{\text{STP}}^{ 3}$ /m mmHg]	3.95	0.58	16.03	6.60	8.80	13.33	21.10	4.83	20.26	1.41

The results showed that the evaluated values of diffusion coefficient differ for water and ethanol permeating through epichlorohydrin-crosslinked chitosan MMMs. Both, pristine epichlorohydrin-crosslinked chitosan membranes and membranes contained Cr_2_O_3_ and TiO_2_, reach much higher value of ethanol diffusion coefficient than for water. Otherwise, for membranes with Fe_3_O_4_ filler the reverse trend in diffusion coefficient was observed.

The addition of iron(II,III) oxide particles influenced both the diffusion and solubility coefficients of water and ethanol. Magnetite presence invoked increasing of water diffusion coefficient and decreasing of ethanol diffusion coefficient in comparison to pristine membrane. Additionally, increasing content of filler influenced further decrease of ethanol diffusion coefficient from 70.1 × 10^−14^ to 22.7 × 10^−14^ m^2^/s, but increased ethanol solubility from 8.80 ${\text{m}}_{\text{STP}}^{3} /{\text{m}}^{ 2}$/m mmHg to 21.10 ${\text{m}}_{\text{STP}}^{3} /{\text{m}}^{2}$/m mmHg. Despite increasing ethanol solubility with higher amount of magnetite, their values are 3.5 times lower than for water, which in effect better penetrate into membrane. Presence of Fe_3_O_4_ particles makes membrane less susceptible to swelling in water which causes a decrease their permeation across the membrane and the observed values of water permeation coefficient decreased.

For pristine chitosan membrane the diffusion coefficients for water and ethanol were equal 8.9 × 10^−14^ and 131.2 × 10^−14^ m^2^/s, respectively. The addition of Cr_2_O_3_ particles into chitosan matrix increased the diffusion coefficient of both feed’s components, i.e. 4.5 and 3.5 times for water and ethanol, respectively. Addition of hydrophilic Cr_2_O_3_ increased the membrane hydrophilicity and increased water transport across this membrane was observed. Furthermore, greater amount of filler creates an extra free volume in polymer matrix, and in consequence, offers more space for permeating both water and ethanol molecules. The highest content of Cr_2_O_3_ (15 wt%) influences increase of water solubility coefficient and ethanol diffusion coefficient, although decreases the ethanol solubility coefficient and water diffusion coefficient.

When polymer matrix contained TiO_2_, decrease of ethanol diffusion coefficient from 131.2 × 10^−14^ to 99.7 × 10^−14^ m^2^/s and considerable increase of water diffusion coefficient from 8.9 × 10^−14^ to 47.3 × 10^−14^ m^2^/s were observed; however, contrary trend of solubility coefficient, i.e. increase of ethanol and decrease of water solubility coefficient were observed. Further addition of TiO_2_ increases both water and ethanol permeation coefficient and decrease their diffusion coefficients, whereas 15 wt% TiO_2_ has influence on increase diffusion coefficients and decrease solubility coefficients for water and ethanol molecules.

Despite the created free volumes, for 15 wt% Cr_2_O_3_ loaded membrane permeation coefficient for both, water and ethanol decrease in similar manner to the membrane containing 15 wt% of TiO_2_ particles.

**Fig. 5:**
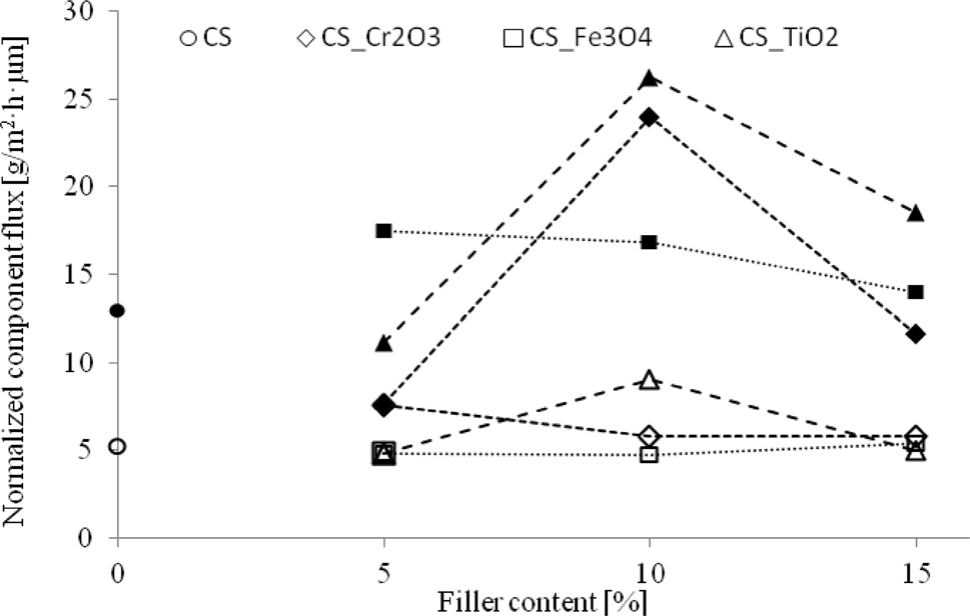
Normalized component’s fluxes for pristine and composite epichlorohydrin-crosslinked chitosan MMMs (filled marks—ethanol, blank marks—water)

The presence of titanium(IV) oxide mainly affected ethanol transport rather than water. Addition of hydrophilic filler raised the membrane hydrophilic character and reduced a membrane affinity to organic solvent. Unfortunately, higher content of this filler caused mostly increasing of ethanol content in permeate rather than water. This phenomenon can be explained by the fact that the addition of TiO_2_ nanoparticles to the chitosan matrix created extra free volumes in polymer, and in consequence, offered space for easier permeation of water molecules through membrane. When filer content was 15 wt%, both ethanol and water fluxes decreased. Similar remarks was observed by Sarinam et al. (2006), where TiO_2_ particles at high content in the PVA matrix will act as the reinforcing bridge elements, thus making the PVA chains more tighter, thereby giving a reduced swelling effect in water and ethanol (Table 2) with the simultaneous decrease in flux at higher amount of TiO_2_ (Fig. 5).

**Table 2:** Degree of swelling in distilled water and pure ethanol (99.8%) measured for pristine epichlorohydrin-crosslinked chitosan membranes and with different oxide filler

Membrane	CS	CS_Cr_2_O_3_			CS_Fe_3_O_4_			CS_TiO_2_		
Filler content [wt%]	0	5	10	15	5	10	15	5	10	15
Degree of swelling in water [%]	108.6	94.9	92.7	121.5	103.9	95.2	89.9	105.4	111.1	96.7
Degree of swelling in ethanol [%]	4.3	5.8	1.1	0.8	1.6	3.5	2.8	5.2	6.2	0.8

Addition of 5 wt% Cr_2_O_3_ to chitosan membrane caused increasing water and decreasing ethanol normalized flux in comparison to membrane without filler. Higher content of Cr_2_O_3_, analogical as for TiO_2_, made more free volume available in membrane and implied higher ethanol normalized flux. Further decreasing of ethanol normalized flux was observed above this content of Cr_2_O_3_, when degree of swelling in water significantly increased and membrane affinity toward ethanol is decreasing.

When 5 wt% of Fe_3_O_4_ was added to the pristine membrane, normalized flux of ethanol increased, but the future filler implementation results in a gradual decrease of ethanol normalized flux. On the other hand, normalized flux of water increased, despite decreased degree of swelling in water, if the content of magnetite in MMMs is increasing. At the beginning addition of Fe_3_O_4_ particles to the MMMs caused an increase of free volume in polymer matrix, but despite the future increase of filler amount, the determined value of a total flux did not increase. This is probably related to the magnetic properties of Fe_3_O_4_—higher amount generate stronger magnetic field, which has beneficial influence on the membrane separation properties constituting a barrier for ethanol molecules while facilitating the transport of water molecules across the membrane. Magnetite, as a filler, affects the composition of the permeate—increasing water and decreasing ethanol normalized fluxes, but not influencing the value of total normalized flux, in effect improve in this way the separation factor of these membranes.

In order to compare the separation efficiency of different investigated membranes, pervaporation separation index was used (Table 3). It can be seen that different metal oxides influence the membrane properties in different ways. Titanium(IV) oxide has a major influence on normalize total flux. Unfortunately, presence of TiO_2_ influence rather on the ethanol flux, which is higher when membrane contained above 5 wt% filler, in effect their separation factor is not impressive. On the other hand, iron(II,III) oxide has influence more on separation factor than on the total normalized flux. Separation factor for membrane containing 15 wt% Fe_3_O_4_ was equal to 16.3 and was about eight times higher than for pristine membrane. Chromium(III) oxide influence on both transport parameters, but filler content level above 10 wt% results in the deterioration of transport characteristics with respect to the pristine membrane. The highest values of pervaporation separation index were obtained for membranes containing 15 wt% Fe_3_O_4_ (296.8 kg/m^2^ h µm), 5 wt% Cr_2_O_3_ (132.2 kg/m^2^·h µm), 10 wt% Cr_2_O_3_ (83.4 kg/m^2^ h µm), 10 wt% Fe_3_O_4_ and 5 wt% Fe_3_O_4_ (79.6 and 75.8 kg/m^2^ h µm, respectively).

**Table 3:** Comparison of pervaporation separation index, PSI for all studied chitosan MMMs

Membrane	CS	CS_Cr_2_O_3_			CS_Fe_3_O_4_			CS_TiO_2_		
Filler content [wt%]	0	5	10	15	5	10	15	5	10	15
Normalized total flux, *J*_*N*_ × 10^3^ [kg/m^2^ h μm]	18.1	15.2	29.8	17.4	22.3	21.5	19.4	16.0	35.3	23.5
Separation factor, *α*_AB_ [−]	1.9	9.7	3.8	1.3	4.4	4.7	16.3	1.6	1.5	1.5
Pervaporative separation index, PSI × 10^3^ [kg/m^2^ h μm]	16.3	132.2	83.4	5.2	75.8	79.6	296.8	9.6	17.7	11.8

### Comparsion of pervaporation performance of chitosan-based hybrid membranes

Chitosan, as a biopolymer, is widely used as a membrane material for pervaporation dehydration of organic–aqueous solutions due to its outstanding selectivity toward water, adhesiveness, film-forming ability, and resistance to organic solvents. Table 4 summarizes the pervaporation performance of chitosan-based homogenous as well as hybrid membranes for the dehydration of ethanol solution reported in the literature. It could be seen that there was a relationship between the flux and the separation factor. Unfortunately, it was very rare to have a high efficiency of the process at high flux values, on the other hand low value of obtained flux made impossible to use this type of membrane on an industrial scale. However, very important factors in the pervaporative dehydration process were temperature and the feed concentration.

**Table 4:** Comparsion of pervaporation performance of chitosan-based membranes for dehydration of ethanol aqueous solution

Polymer matrix	Filler/content (wt%)	Cross-linking agent	Ethanol in feed (wt%)	Temp (°C)	Flux (kg/m^2^ h)	Separation factor α (−)	References
Chitosan	–	–	96	40	0.007	202	(Uragami and Takigawa 1990)
Chitosan	–	–	85	50	0.275	200	(Chen et al. 2007)
Chitosan	–	–	90	30	0.037	41	(Zielinska et al. 2011)
Chitosan	–	–	90	80	0.054	158.02	(Sun et al. 2008)
Chitosan acetate salt	–	–	96	40	0.002	2556	(Uragami and Takigawa 1990)
Chitosan acetate salt	–	–	90	25	0.142	242	(Lee and Shin 1991; Lee 1993)
Chitosan	–	GA	96	40	0.004	2208	(Uragami and Takigawa 1990)
Chitosan	–	GA	90	50	0.201	127	(Zhang et al. 2007)
Chitosan	–	GA	90	60	0.250	105	(Zhang et al. 2007)
Chitosan	–	GA	90	30	0.051	27	(Zielinska et al. 2011)
Chitosan	–	SA	90	60	0.472	1791	(Ge et al. 2000)
Chitosan	–	PA	95.58		0.58	213	(Sunitha et al. 2012)
Chitosan	8% H-ZSM-5	–	90	80	0.231	152.82	(Sun et al. 2008)
Chitosan	6% TiO_2_	–	90	80	0.340	196	(Yang et al. 2009)
Chitosan	6% ZIF-7	GA	90	25	0.337	2368	(Kang et al. 2013)
Chitosan	30% PB^a^	GA	90	25	0.650	1500	(Wu et al. 2016)
Phosphorylated chitosan	–	–	90	70	0.180	541	(Lee and Shin 1991; Lee 1993)
Chitosan/3-aminopropyl-triethoxysilane (10%)	–	–	85	50	0.887	597	(Chen et al. 2007)
Carboxymethylated chitosan	–	–	90	25	0.036	1294	(Lee and Shin 1991, Lee 1993)
Carboxyethylated chitosan	–	–	90	25	0.030	301	(Lee and Shin 1991; Lee 1993)
Cyanoethylated chitosan	–	–	90	25	0.080	52	(Lee and Shin 1991; Lee 1993)
Sulphonated chitosan	–	GA	90	25	0.052	1560	(Lee and Shin 1991; Lee 1993)
Carboxylated chitosan	–	GA/MA	90	50	0.238	991	(Zhang et al. 2007)
Carboxylated chitosan	–	GA/MA	90	60	0.300	634	(Zhang et al. 2007)
Chitosan/hydroxyethylcellulose (3:1)	–	UFSA	90	60	0.112	10.491	(Chanachai et al. 2000)
Chitosan/sodium alginate	–	–	95		0.070	1110	(Moon et al. 1999)
Chitosan/sodium alginate	–	–	86,4		0.220	436	(Kanti et al. 2004)
PVA/chitosan (60/40)	–	GA	90	60	0.47	450	(Lee et al. 1992)
Chitosan	–	ECH	96	25	1.036	1.9	Present work
Chitosan	5% Cr_2_O_3_	ECH	96	25	0.848	9.7	Present work
Chitosan	10% Cr_2_O_3_	ECH	96	25	1.739	3.8	Present work
Chitosan	15% Cr_2_O_3_	ECH	96	25	0.958	1.3	Present work
Chitosan	5% TiO_2_	ECH	96	25	0.878	1.6	Present work
Chitosan	10% TiO_2_	ECH	96	25	1.456	1.5	Present work
Chitosan	15% TiO_2_	ECH	96	25	1.251	1.5	Present work
Chitosan	5% Fe_3_O_4_	ECH	96	25	1.111	4.4	Present work
Chitosan	10% Fe_3_O_4_	ECH	96	25	1.070	4.7	Present work
Chitosan	15% Fe_3_O_4_	ECH	96	25	0.845	16.3	Present work

*GA* glutaraldehyde, *GA/MA* glutaraldehyde and maleic anhydride, *SA* sulfuric acid (VI), *PA* phosphoric acid (V), *F* formaldehyde, *ECH* epichlorohydrin, *UFSA* crosslinking bath containing: urea, formaldehyde and sulfuric acid (VI)

^a^Prussian blue

Changes in the composition of the feed significantly affect the efficiency of the process using hydrophilic membranes, since lowering the water content reduces the possibility of swelling of the membranes and decreasing of permeation flux. Won et al. (1996) studied the influence of feed composition on water transport across pristine chitosan membranes in pervaporation process at 40 °C. They noticed, that the higher concentration of water in separation mixture influences on the increasing of obtained permeation flux. Additionally, the content of water in permeate was not linear. Additionally, increasing the feed temperature also has a positive influence on permeation flux, whereas decreased the separation factor (Jiraratananon et al. 2002).

Membranes prepared in this work are characterized by quite high values of permeation fluxes with much lower values of separation factor than other mixed-matrix membranes presented in literature; however, addition of inorganic filler could improve membrane properties. Furthermore, knowledge on the effect of the type and amount of fill will allow to design new, more efficient membranes for pervaporative dehydration processes.

## Conclusions

In this paper was discussed the influence of filler type and amount presence on the epichlorohydrin-crosslinked chitosan membrane’s water/ethanol separation properties.

In pervaporation experiments as polymer matrix fillers were used particles like Cr_2_O_3_, TiO_2_ and Fe_3_O_4_ content ranged from 5 to 15 wt%. Each filler influences membrane properties in different ways. Magnetite, as a filler affects separation factor and normalized total flux increasing their values. Titanium(IV) oxide affects mainly total normalized flux, but does not change their separation properties, whereas membranes containing chromium(III) oxide influences both, separation factor and normalized total flux.

Comparing the separation properties it can be concluded that the epichlorohydrin-crosslinked chitosan membranes containing iron(II,III) oxide have better separation properties than the corresponding pristine and other prepared membranes. The best separation parameters were obtained for epichlorohydrin-crosslinked chitosan membrane containing 15 wt % Fe_3_O_4_ and 5 wt% Cr_2_O_3_, while membranes containing TiO_2_ had worse properties than pristine membrane.
